# Agonist-Evoked Increases in Intra-Platelet Zinc Couple to Functional Responses

**DOI:** 10.1055/s-0038-1676589

**Published:** 2018-12-31

**Authors:** Niaz S. Ahmed, Maria E. Lopes Pires, Kirk A. Taylor, Nicholas Pugh

**Affiliations:** 1 School of Life Sciences, Anglia Ruskin University, Cambridge, United Kingdom; 2 Cardio-Respiratory Interface Section, National Heart and Lung Institute, Imperial College London, London, United Kingdom

**Keywords:** platelets, zinc, platelet activation, signal transduction, secretory vesicles, granule release

## Abstract

**Background** Zinc (Zn ^2+^ ) is an essential trace element that regulates intracellular processes in multiple cell types. While the role of Zn ^2+^ as a platelet agonist is known, its secondary messenger activity in platelets has not been demonstrated.

**Objectives** This article determines whether cytosolic Zn ^2+^ concentrations ([Zn ^2+^ ] _i_ ) change in platelets in response to agonist stimulation, in a manner consistent with a secondary messenger, and correlates the effects of [Zn ^2+^ ] _i_ changes on activation markers.

**Methods** Changes in [Zn ^2+^ ] _i_ were quantified in Fluozin-3 (Fz-3)-loaded washed, human platelets using fluorometry. Increases in [Zn ^2+^ ] _i_ were modelled using Zn ^2+^ -specific chelators and ionophores. The influence of [Zn ^2+^ ] _i_ on platelet function was assessed using platelet aggregometry, flow cytometry and Western blotting.

**Results** Increases of intra-platelet Fluozin-3 (Fz-3) fluorescence occurred in response to stimulation by cross-linked collagen-related peptide (CRP-XL) or U46619, consistent with a rise of [Zn ^2+^ ] _i_ . Fluoresence increases were blocked by Zn ^2+^ chelators and modulators of the platelet redox state, and were distinct from agonist-evoked [Ca ^2+^ ] _i_ signals. Stimulation of platelets with the Zn ^2+^ ionophores clioquinol (Cq) or pyrithione (Py) caused sustained increases of [Zn ^2+^ ] _i_ , resulting in myosin light chain phosphorylation, and cytoskeletal re-arrangements which were sensitive to cytochalasin-D treatment. Cq stimulation resulted in integrin α _IIb_ β _3_ activation and release of dense, but not α, granules. Furthermore, Zn ^2+^ -ionophores induced externalization of phosphatidylserine.

**Conclusion** These data suggest that agonist-evoked fluctuations in intra-platelet Zn ^2+^ couple to functional responses, in a manner that is consistent with a role as a secondary messenger. Increased intra-platelet Zn ^2+^ regulates signalling processes, including shape change, α _IIb_ β _3_ up-regulation and dense granule release, in a redox-sensitive manner.

## Introduction

Zinc (Zn ^2+^ ) is an essential trace element, serving as a co-factor for 10 to 15% of proteins encoded within the human genome. 1 It is acknowledged as an extracellular signalling molecule in glycinergic and GABAergic neurones, and is released into the synaptic cleft following excitation. 2 3 Zn ^2+^ is concentrated in atherosclerotic plaques and released from damaged epithelial cells, and is released from platelets along with their α-granule cargo following collagen stimulation. 4 Therefore, increased concentrations of unbound or labile (free) Zn ^2+^ are likely to be present at areas of haemostasis, and may be much higher in the microenvironment of a growing thrombus. Zn ^2+^ plays a role in haemostasis by contributing to wound healing, 5 and regulating coagulation, for example, as a co-factor for factor XII. 6 Labile Zn ^2+^ acts as a platelet agonist, being able to induce tyrosine phosphorylation, integrin α _IIb_ β _3_ activation and aggregation at high concentrations, while potentiating platelet responses to other agonists at lower concentrations. 7 Zn ^2+^ is directly linked to platelet function *in vivo* , as dietary Zn ^2+^ deficiency of humans or rodents manifests with a bleeding phenotype that reverses with Zn ^2+^ supplementation.

Labile, protein-bound and membrane-bound, Zn ^2+^ pools are found within multiple cell types, including immune cells and neurones. These pools are inter-changeable, allowing increases in the bioavailability of Zn ^2+^ to Zn ^2+^ -sensitive proteins following signalling-dependent processes. In this manner, Zn ^2+^ is acknowledged to behave as a secondary messenger. 8 In nucleated cells, Zn ^2+^ is released from intracellular granules into the cytosol via Zn ^2+^ transporters, or from Zn ^2+^ -binding proteins such as metallothioneins, following engagement of extracellular receptors. For example, a role for Zn ^2+^ as a secondary messenger has been shown in mast cells, where engagement of the F _C_ ε receptor I results in rapid increases in intracellular Zn ^2+^ (Zn ^2+^ ] _i_ ). This ‘zinc wave’ modulates transcription of cytokines, and affects tyrosine phosphatase activity. 8 Zn ^2+^ also acts as a secondary messenger in monocytes, where stimulation with lipopolysaccharide results in increases in [Zn ^2+^ ] _i_ , suggestive of a role in transmembrane signalling. 9 Agonist-evoked changes of [Zn ^2+^ ] _i_ modulate signalling proteins (i.e. protein kinase C [PKC], calmodulin-dependent protein kinase II [CamKII] and interleukin receptor-associated kinase) in a similar manner to calcium (Ca ^2+^ )-dependent processes. 4 8 10 While the role of Zn ^2+^ as a secondary messenger in nucleated cells has gathered support in recent years, agonist-dependent regulation of [Zn ^2+^ ] _i_ in platelets during thrombosis has yet to be demonstrated.

Here, we utilize Zn ^2+^ -specific fluorophores, chelators and ionophores to investigate the role of [Zn ^2+^ ] _i_ fluctuations in platelet behaviour. We show that agonist-evoked elevation of [Zn ^2+^ ] _i_ regulates platelet shape change, dense granule release and phosphatidylserine (PS) exposure. These findings indicate a role for Zn ^2+^ as a secondary messenger, which may have implications for the understanding of platelet signalling pathways involved in thrombosis during adverse cardiovascular events.

## Experimental Procedures

*Materials* : Fluozin-3- am (Fz-3, Zn ^2+^ indicator) and Fluo-4- am (Ca ^2+^ indicator) were from Invitrogen (Paisley, United Kingdom). Z-VAD (pan-caspase inhibitor) was from R&D Systems (Abingdon, United Kingdom). Primary anti-vasodilator-stimulated phosphoprotein (VASP) (Ser157) and anti-myosin light chain (MLC) (Ser19) antibodies were from Cambridge Bioscience (Cambridge, United Kingdom), and fluorescently conjugated procaspase-activating compound 1 (PAC-1), CD62P and CD63 antibodies were from BD Biosciences (Oxford, United Kingdom). Cross-linked collagen-related peptide (CRP-XL; glycoprotein VI [GpVI] agonist) was from Professor Richard Farndale (Cambridge, United Kingdom), U46619 (thromboxane [TP]α receptor agonist) was from Tocris (Bristol, United Kingdom), thrombin (protease-activated receptor [PAR] agonist) was from Sigma Aldrich (Poole, United Kingdom) and cytochalasin-D (Cyt-D, actin polymerization inhibitor) was from AbCam (Cambridge, United Kingdom). Clioquinol (Cq, Zn ^2+^ ionophore, C _9_ H _5_ ClINO, K _d_ Zn: 10 ^−7^ M, K _d_ Ca: 10 ^−4.9^ M), pyrithione (Py, Zn ^2+^ ionophore, C _10_ H _8_ N _2_ O _2_ S _2_ , K _d_ Zn: 10 ^−7^ M, K _d_ Ca: 10 ^−4.9^ M), A23187 (Ca ^2+^ ionophore, C _29_ H _37_ N _3_ O _6_ ), N,N,N′,N′-Tetrakis(2-pyridylmethyl)ethylenediamine (TPEN, Zn ^2+^ chelator, K _d_ Zn: 2.6 × 10 ^−16^ M, K _d_ Ca: 4.4 × 10 ^−5^ M, 11 12 13 14 ), dimethyl-bis-(aminophenoxy)ethane-tetraacetic acid (DM-BAPTA)-AM (C _34_ H _40_ N _2_ O _18_ , K _d_ Zn: 7.9 × 10 ^−9^ M, K _d_ Ca: 110 × 10 ^−9^ M, 11 12 13 14 ) and membrane permeant anti-oxidizing proteins, polyethylene glycol-superoxide dismutase (PEG-SOD) and PEG-catalase (CAT) were from Sigma Aldrich. Unless stated, all other reagents were from Sigma Aldrich.

*Preparation of washed human platelets* : This study was approved by the Research Ethics Committee at Anglia Ruskin University and informed consent was obtained in accordance with the Declaration of Helsinki. Blood was donated by healthy human volunteers, free from medication for 2 weeks. Blood was collected into 11 mM sodium citrate and washed platelets were prepared as described previously. 7 Unless otherwise stated, to isolate the mechanisms of Zn ^2+^ fluctuations from other cation-specific effects, experiments were performed in the absence of extracellular Ca ^2+^ .

*Cation mobilisation studies* : For studies of [Zn ^2+^ ] _i_ or [Ca ^2+^ ] _i_ mobilization, platelet-rich plasma was loaded with Fz-3 (2 µM, 30 minutes, 37°C), or Fluo-4 (2 µM, 30 minutes, 37°C). Fz-3 is responsive to Zn ^2+^ in the nM range and is not significantly affected by Ca ^2+^_._ 15 Platelets were collected by centrifugation (350 × *g* , 15 minutes), re-suspended in Ca ^2+^ -free Tyrode's buffer (in mM: 140 NaCl, 5 KCl, 10 HEPES, 5 glucose, 0.42 NaH _2_ PO _4_ , 12 NaHCO _3_ , pH 7.4) and rested at 37°C for 30 minutes prior to use. Fluorescence was monitored using a Fluoroskan Ascent fluorometer (ThermoScientific, United Kingdom) using 488 nm and 538 nm excitation and emission filters, respectively. Washed Fz-3 or Fluo-4 loaded platelet suspensions were treated with ionophores or chelators to calibrate *R*_max_ or *R*_min_ values ( Supplementary Fig. S1 , available in the online version). Results are expressed as an increase of background-corrected fluorescence at each time point relative to baseline: ( *F* - *F*_background_ )/ *F*_0_ - *F*_background_ ).

*Optical aggregometry* : Aggregometry was performed with washed platelet suspensions under stirring conditions at 37°C in an AggRam light transmission aggregometer (Helena Biosciences, Gateshead, United Kingdom). 7 Aggregation traces were acquired using a proprietary software (Helena Biosciences) and analysed within GraphPad Prism (Version 6.03).

*Confocal microscopy* : Images of platelets adhering to coated fibrinogen coverslips (100 µM) were acquired using a LSM510/Axiovert laser scanning confocal microscope with 60× oil NA1.45 objective (Zeiss, United Kingdom). Surface coverage of DIOC _6_ -stained platelets was quantified using ImageJ (v1.45, National Institutes of Health, Bethesda, Maryland, United States).

*Western blotting* : Western blotting was performed as described previously. 7 Briefly, polyvinylidene difluoride membranes were incubated with MLC (1:400) or VASP (Ser157, 1:400) primary antibodies, and horseradish peroxidase-conjugated secondary antibodies (1:7,500).

*Flow cytometry* : Washed platelet suspensions were incubated with fluorescently conjugated antibodies targeting markers of platelet activation: PAC-1 (α _IIb_ β _3_ activation), CD62P (α granule release) and CD63 (dense granule release). Antibody binding following agonist or ionophore stimulation was assessed using an Accuri C6 flow cytometer (BD Biosciences).

*Data analysis* : Maximum and minimum aggregation and *F* / *F*_0_ values were calculated using Microsoft Excel. Western blots were analysed using ImageJ. Data were analysed in GraphPad Prism by two-way analysis of variance followed by Tukey's post hoc test. Significance is denoted as *** *p* < 0.001, ** *p* < 0.01 or * *p* < 0.05.

## Results

**Fig. 1:**
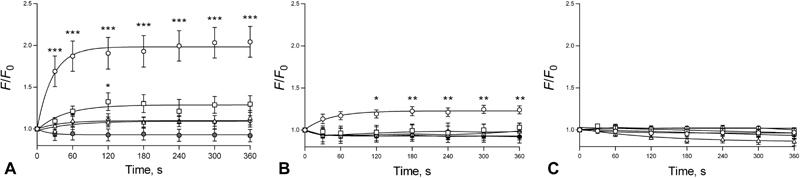
Agonist-dependent platelet activation via GpVI or TP, but not PARs elevates [Zn ^2+^ ] _i_ . Fz-3-labelled washed human platelets were stimulated by CRP-XL ( **A** ), U46619 ( **B** ) or thrombin ( **C** ) and [Zn ^2+^ ] _i_ fluctuations were monitored over 6 minutes using fluorometry. ( **A** ) Fz-3 responses to ○ 1 µg/mL, □ 0.3 µg/mL, ▵ 0.1 µg/mL, ⋄ 0.03 µg/mL CRP-XL or • vehicle (DMSO). ( **B** ) Fz-3 responses to ○ 10 µM, □ 3 µM, ▵ 1 µM, ⋄ 0.3 µM U46619 or • vehicle (DMSO). ( **C** ) Fz-3 responses to, ○ 1 U/mL, □ 0.3 U/mL, ▵ 0.1 U/mL, ⋄ 0.03 U/mL thrombin or • vehicle (DMSO). Data are mean ± standard error of the mean (SEM) from at least 8 independent experiments. Significance is denoted as *** *p* < 0.001, ** *p* < 0.01 or * *p* < 0.05.

[Zn ^2+^ ] _i_ fluctuations coordinate receptor stimulation with signalling responses in nucleated cells. 8 To investigate whether intra-platelet Zn ^2+^ fluctuates during activation, agonist-evoked changes of [Zn ^2+^ ] _i_ were monitored in washed platelet suspensions, loaded with the Zn ^2+^ -specific fluorophore, Fz-3. Stimulation with conventional platelet agonists CRP-XL and U46619 induced rapid, dose-dependent increases of fluorescence peaking after approximately 2 minutes, consistent with increases in [Zn ^2+^ ] _i_ . At 6 minutes, 1 µg/mL CRP-XL or 10 µM U46619 stimulation increased *F* / *F*_0_ to 2.0 ± 0.1 and 1.2 ± 0.1 AU, respectively (compared with 0.9 ± 0.2 AU for the vehicle control, *p* < 0.05, Fig. 1A , B ). Conversely, thrombin stimulation did not elevate Fz-3 fluorescence ( Fig. 1C ). These data indicate that platelet activation via GpVI and TP, but not via PARs, leads to signalling responses that result in the elevation of [Zn ^2+^ ] _i_ , in a similar manner to agonist-evoked increases in [Ca ^2+^ ] _i_ . Inclusion of 2 mM CaCl _2_ in the extracellular medium did not significantly affect agonist-evoked responses ( Supplementary Fig. S2 , available in the online version).

**Fig. 2:**
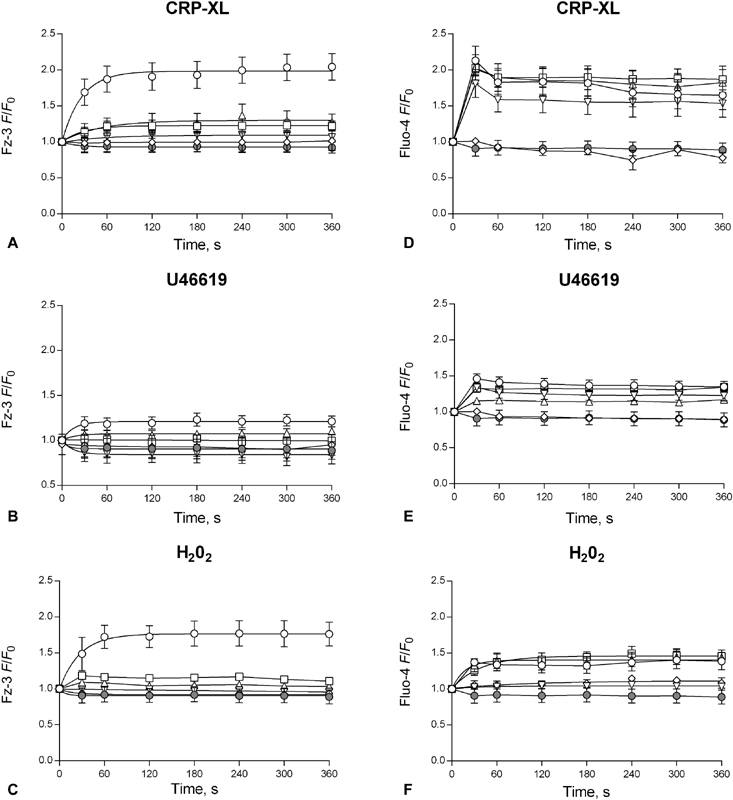
Agonist-dependent intracellular zinc ([Zn ^2+^ ] _i_ ) fluctuations are sensitive to the platelet redox state. Platelets were loaded with Fz-3 ( **A** , **B** , **C** ), or Fluo-4 ( **D** , **E** , **F** ) and stimulated with CRP-XL (1 µg/mL, ○, **A** , **D** ), U46619 (10 µM, ○ **B** , **E** ) or H _2_ O _2_ (10 µM, ○, **C** , **F** ), during which changes in fluorescence were monitored. Where indicated, platelets were pre-treated with TPEN (▿, 50 µM), DM-BAPTA (⋄, 10µM), PEG-SOD (□, 30 U/mL), PEG-CAT (▵, 300 U/mL) or vehicle (DMSO), •). Data are mean ± standard error of the mean (SEM) from at least 5 independent experiments. Significance is denoted as *** *p* < 0.001, ** *p* < 0.01 or * *p* < 0.05.

Experiments were performed to confirm the specificity of fluorescence fluctuations for Zn ^2+^ . Platelets were pre-treated with the intracellular Zn ^2+^ -specific chelator TPEN (50 µM) prior to stimulation with 1 µg/mL CRP-XL. Fz-3 responses were reduced to 1.1 ± 0.1 AU, compared with of 2.0 ± 0.1 AU for CRP-XL stimulation alone ( *p* < 0.05, Fig. 2A ). Interestingly, treatment with DM-BAPTA (10 µM), a non-specific cation chelator, led to a similar reduction (to 1.0 ± 0.1 AU, *p* < 0.05). Abrogation of Fz-3 fluorescence was also observed following stimulation with U46619 (10 µg/mL), where TPEN or DM-BAPTA treatment reduced *F* / *F*_0_ plateau levels from 1.2 ± 0.1 to 0.8 ± 0.1 AU and 1.0 ± 0.1 AU, respectively ( *p* < 0.05, Fig. 2B ). Further experiments were performed to investigate the influence of cation chelation on [Ca ^2+^ ] _i_ fluctuations using Fluo-4-loaded platelets. As previously demonstrated, CRP-XL- and U46619-induced Ca ^2+^ signals were absent following BAPTA treatment ( *F* / *F*_0_ signals were reduced from 1.6 ± 0.2 to 0.8 ± 0.1 AU, and from 1.4 ± 0.1 to 0.9 + 0.0 AU, for CRP-XL and U46619 stimulation, respectively, *p* < 0.05, Fig. 2D , E ). However, Fluo-4 fluorescence was not significantly affected by TPEN treatment (1.5 ± 0.2 and 1.2 ± 0.1 AU for CRP-XL and U46619, respectively, ns) indicating that TPEN does not chelate [Ca ^2+^ ] _i_ , and that Fz-3 signals are attributable to [Zn ^2+^ ] _i_ with no influence from other cations. Furthermore, these data demonstrate that fluctuations in [Zn ^2+^ ] _i_ do not affect agonist-evoked Ca ^2+^ signals.

Agonist-evoked [Zn ^2+^ ] _i_ increases may result from release of membrane-bound intracellular stores or by liberation from metal-binding proteins (e.g. metallothioneins) in response to redox-mediated modifications to thiol groups. 16 To investigate the nature of the Zn ^2+^ source, platelets were treated with membrane-permeant anti-oxidizing proteins PEG-SOD and PEG-CAT, 17 and CRP-XL-evoked [Zn ^2+^ ] _i_ fluctuations were monitored. PEG-SOD and PEG-CAT both abolished CRP-XL-induced increases of Fz-3 fluorescence, indicating redox-dependent modulation of Zn ^2+^ release (PEG-SOD and PEG-CAT reduced *F* / *F*_0_ plateaus following 1 µg/mL CRP-XL treatment from 2.0 ± 0.1 to 1.2 ± 0.1 AU and 1.3 ± 0.1 AU, respectively, *p* < 0.05, Fig. 2A ). This is consistent with published data showing a greater capacity for GpVI to influence redox signalling than other receptors. 18 Similarly, PEG-SOD and PEG-CAT abolished U46619-induced [Zn ^2+^ ] _i_ responses (to 1.0 ± 0.0 and 1.1 ± 0.0 AU, respectively, following 10 µM U46619 stimulation, *p* < 0.05, Fig. 2B ). PEG-SOD and PEG-CAT did not affect CRP-XL- or U46619-mediated Fluo-4 fluorescence, suggesting that [Zn ^2+^ ] _i_ but not [Ca ^2+^ ] _i_ signals are regulated by redox-sensitive processes.

Further experiments were performed to resolve the relationship between the platelet redox state and [Zn ^2+^ ] _i_ fluctuations. Treatment with H _2_ O _2_ mimics increases in platelet reactive oxygen species (ROS). 19 H _2_ O _2_ increased both [Ca ^2+^ ] _i_ and [Zn ^2+^ ] _i_ ( *F* / *F*_0_ plateaus were 1.8 + 0.3 AU following H _2_ O _2_ [10 µM] stimulation of Fz3-loaded platelets, compared with 0.9 ± 0.1 AU for vehicle-treated platelets, while H _2_ O _2_ stimulation increased Fluo-4 fluorescence from 0.9 ± 0.1 to 1.4 ± 0.1 AU, *p* < 0.05, Fig. 2C , F ). H _2_ O _2_ -mediated [Zn ^2+^ ] _i_ increases were abrogated with PEG-SOD or PEG-CAT, while [Ca ^2+^ ] _i_ was unaffected ( Fig. 2E , F ). These data support a role for the platelet redox state in regulating [Zn ^2+^ ] _i_ fluctuations.

**Fig. 3:**
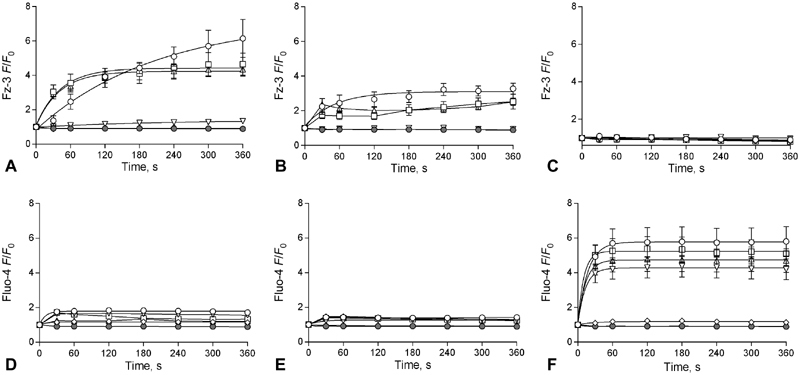
Treatment of platelets with Zn ^2+^ ionophores clioquinol (Cq) or pyrithione (Py) elevates [Zn ^2+^ ] _i_ , but not [Ca ^2+^ ] _i_ . Washed platelet suspensions were loaded with Fz-3 ( **A** , **B** , **C** ), or Fluo-4 ( **D** , **E** , **F** ) and stimulated with Cq (○, 300 µM, **A** , **D** ), Py (○, 300 µM, **B** , **E** ) or A23187 (○, **C** , **F** ). Where indicated, platelets were pre-treated with (TPEN) (50 µM, ▿), DM-BAPTA (10 µM, ⋄), PEG-SOD (30 U/mL, □), PEG-CAT (300 U/mL, ▵), or vehicle (DMSO), •. Data are mean ± standard error of the mean (SEM) from at least 6 independent experiments. Significance is denoted as *** *p* < 0.001, ** *p* < 0.01 or * *p* < 0.05.

Having demonstrated that intra-platelet Zn ^2+^ rises in response to agonist stimulation, we further examined the influence of [Zn ^2+^ ] _i_ on platelet responses. We hypothesized that liberation of Zn ^2+^ from intracellular stores (such as platelet α-granules 20 ) using specific ionophores would result in increased [Zn ^2+^ ] _i_ , in a similar manner A23187-evoked Ca ^2+^ responses. 21 Zn ^2+^ ionophores Cq and Py have previously been used to model [Zn ^2+^ ] _i_ increases in nucleated cells. 22 23 24 We utilized these reagents to model agonist-evoked [Zn ^2+^ ] _i_ increases in washed platelet suspensions. Stimulation with Cq or Py produced large elevations of [Zn ^2+^ ] _i_ , with *F* / *F*_0_ plateaus of 7.9 ± 0.5 and 3.3 ± 0.3 AU, respectively ( *p* < 0.05, Fig. 3A , B ). The extent of [Zn ^2+^ ] _i_ increase was greater than that observed following CRP-XL stimulation, suggesting that liberation from stores is not the principal means by which [Zn ^2+^ ] _i_ increases following agonist stimulation. Zn ^2+^ ionophore-dependent Fz-3 fluorescence increases were sensitive to pre-treatment with TPEN or BAPTA, consistent with a role for Cq or Py increasing [Zn ^2+^ ] _i_ ( Fig. 3A , B ). However, [Zn ^2+^ ] _i_ signals were not influenced by PEG-SOD or PEG-CAT, demonstrating that ionophore-induced [Zn ^2+^ ] _i_ release is not redox sensitive. Cq or Py stimulation did not affect Fluo-4 fluorescence ( Fig. 3D , E ), indicating that Zn ^2+^ ionophores have a negligible affinity for Ca ^2+^ . A23187 increased Fluo-4 fluorescence (from 0.9 ± 0.1 to 5.8 ± 0.9 AU after 6 minutes, *p* < 0.05, Fig. 3F ), but had no effect on Fz-3 fluorescence ( Fig. 3C ), demonstrating that Fz-3 fluorescence is not affected by changes in [Ca ^2+^ ] _i_ . In a similar manner to agonist-dependent Ca ^2+^ signalling, A23187-dependent [Ca ^2+^ ] _i_ increases were abrogated by BAPTA, but were unaffected by TPEN. Thus, Fluo-4 fluorescence is not influenced by Zn ^2+^ .

**Fig. 4:**
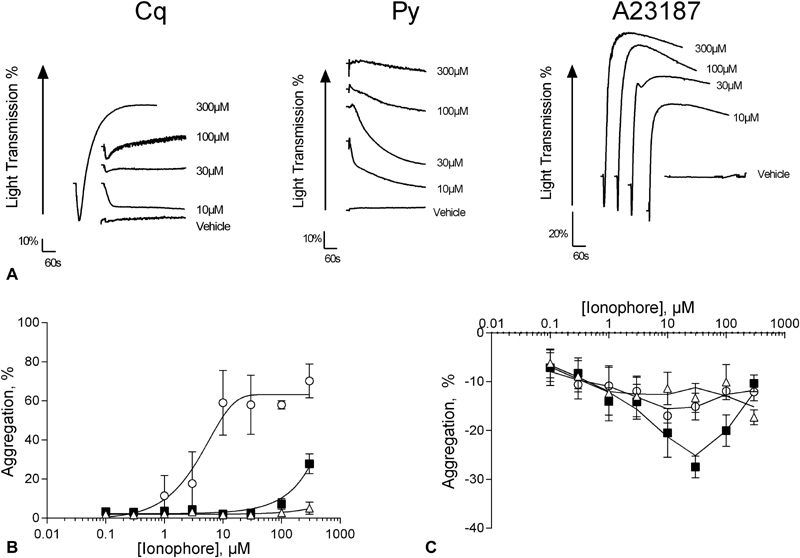
Stimulation of platelets with Zn ^2+^ ionophores leads to shape change. ( **A** ) Washed platelet suspensions were stimulated with different concentrations of clioquinol (Cq), pyrithione (Py) or A23187 during which changes in light transmission were monitored using optical aggregometry. Initial downward deflections indicate a reduction in light transmission that are consistent with shape change. Subsequent upward deflections indicate increases in light transmission, consistent with platelet aggregation. The maximum ( **B** ) and minimum ( **C** ) extent of aggregation were calculated for each ionophore (▪ Cq, ▵ Py, ○ A23187). Data are mean ± standard error of the mean (SEM) from at least 5 experiments.

Our data confirm that platelet [Zn ^2+^ ] _i_ increases can be modelled using the Zn ^2+^ ionophores Cq and Py. Next, we examined the influence of increases in [Zn ^2+^ ] _i_ on platelet aggregation. High concentrations of Cq (300 µM) resulted in an initial decrease in light transmission, followed by a substantial increase, consistent with shape change and aggregation. Platelet aggregates were present following visual inspection of test cuvettes at the end of each experiment (not shown). The extent of Cq-induced aggregation (300 µM, 27.8 ± 5.0%) was lower than that for A23187 (300 µM, 70.2 ± 8.6%, *p* < 0.05, Fig. 4A , B ). Treatment with lower concentrations of Cq (30 µM) resulted in shape change only, with no progression to aggregation. Py stimulation did not cause aggregation but did result in shape change ( Fig. 4A – C ). Response to Py were biphasic, with intermediate concentrations (10–30 µM) resulting in shape change, and higher concentrations having no effect.

The degree of shape change was quantified by calculating the lowest light transmission during ionophore-induced aggregation (denoted minimum aggregation, %). Shape change following Cq or A213817 treatment was comparable (minimum aggregation for 30 µM Cq or Py was –13.3 ± 2.9 and –27.5 ± 2.2%, respectively, compared with –15.1 ± 2.7% for 30 µM A23187, ns, Fig. 4C ). These data are consistent with a role for [Zn ^2+^ ] _i_ in regulating cytoskeletal changes in a similar manner to [Ca ^2+^ ] _i_ -induced shape change.

**Fig. 5:**
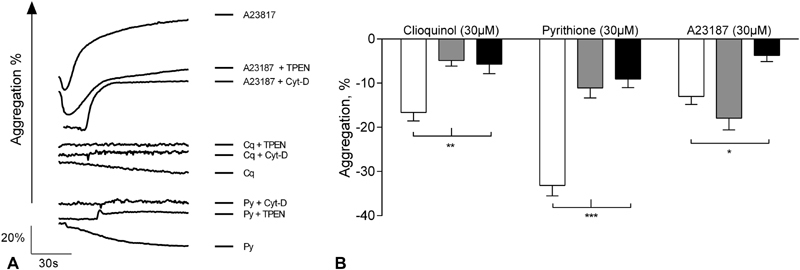
Ionophore-induced shape change is sensitive to pre-treatment with (Cyt-D) or TPEN. ( **A** ) Representative aggregometry traces showing clioquinol (Cq)-, pyrithione (Py)- or A23187-induced (30 µM) shape change following pre-treatment with TPEN (50 µM) or Cyt-D (10 µM). ( **B** ) Quantitation of minimum aggregation following treatment of platelets pre-treated with TPEN (▪ 25 µM), Cyt-D (▪ 10 µM) or vehicle (□ DMSO, prior to stimulation with Cq, Py or A23187 (30 µM). Data are mean ± standard error of the mean (SEM) of at least 6 experiments. Significance is denoted as *** *p* < 0.001, ** *p* < 0.01 or * *p* < 0.05.

To confirm that the changes in light transmission were a biological, rather than chemical phenomenon, we took a pharmacological approach by pre-treating platelets with the actin polymerization inhibitor Cyt-D prior to ionophore stimulation. Cyt-D abrogated Cq-, Py- and A23187-induced shape change, consistent with a genuine biological effect. The minimum aggregation for Cyt-D treated and untreated platelets were –5.7 ± 2.1 and –16.7 ± 1.9%, respectively, following Cq stimulation, –9.1 ± 1.9 and –33.2 ± 2.4, respectively, following Py stimulation, and –3.7 ± 1.4 and –13.0 ± 1.8%, respectively, following A23187 stimulation (30 µM, *p* < 0.05, Fig. 5A , B ). Pre-treatment of platelets with TPEN abrogated Cq- or Py-induced shape change but had no effect on A23187 treatment (minimum aggregation following TPEN treatment was –4.9 ± 1.2, –11.1 ± 2.3 and –17.9 ± 2.6% for Cq, Py and A23817, respectively, *p* < 0.05, Fig. 5A , B ). These data are consistent with a role for [Zn ^2+^ ] _i_ in regulating cytoskeletal re-arrangements. The resistance of A23187-induced shape change to TPEN treatment suggests that the contribution of Ca ^2+^ signals to cytoskeletal re-arrangement occurs independently of Zn ^2+^ signals, and could indicate different mechanisms for Zn ^2+^ - and Ca ^2+^ -induced shape change.

**Fig. 6:**
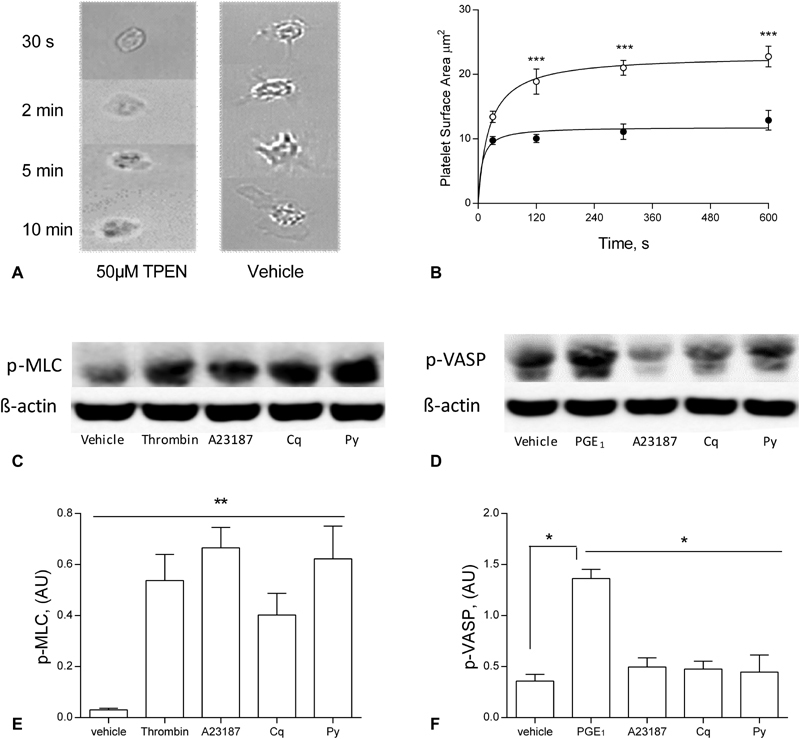
[Zn ^2+^ ] _i_ regulates platelet shape change, and phosphorylation of cytoskeletal regulators. Washed platelet suspensions were incubated on fibrinogen-coated coverslips following pre-treatment with 50 µM TPEN or vehicle control (DMSO). ( **A** ) Representative images of platelet spreading. ( **B** ) Quantification of the surface coverage by adherent platelets (○ DMSO, • 50 µM TPEN, *n* = 3). ( **C** ) Representative Western blot showing increased MLC phosphorylation following stimulation of platelets for 2 minutes with vehicle (DMSO), thrombin (1 U/mL), A23187 (100 µM), clioquinol (Cq) (300 µM) and pyrithione (Py) (300 µM). ( **D** ) Representative Western blot showing VASP phosphorylation following stimulation of platelets for 2 minutes with vehicle (DMSO), prostaglandin E _1_ (PGE _1_ ) (1 U/mL), A23187 (100 µM), Cq (300 µM) and Py (300 µM). VASP phosphorylation was unaffected by Zn ^2+^ ionophore treatment. Blots are representative of three experiments. Data are means ± standard error of the mean (SEM), from at least 5 independent experiments. Significance is denoted as *** *p* < 0.001, ** *p* < 0.01 or * *p* < 0.05.

[Zn ^2+^ ] _i_ -dependent cytoskeletal changes were further investigated by visualizing platelet spreading on fibrinogen. TPEN-treated platelets were able to adhere to fibrinogen, but did not spread, with no visible lamellipodia or filopodia ( Fig. 6A ). Mean platelet surface coverage after 10 minutes was 12.8 ± 1.5 µm, compared with 22.7 ± 1.6 µm for untreated platelets ( Fig. 6B ). Regulation of Cq-induced shape change was investigated by assaying VASP and MLC, which alter phosphorylation status during cytoskeletal re-arrangements. 25 26 Cq- or Py-induced shape change were accompanied by increased phosphorylation of ser157 of MLC, confirming a role for [Zn ^2+^ ] _i_ in the signalling process leading to cytoskeletal changes. Unlike PGE _1_ treatment, VASP did not undergo phosphorylation in response to ionophore treatment, indicating that Zn ^2+^ does not influence activity of cyclic nucleotide-dependent kinases such as protein kinase A (PKA) or protein kinase G (PKG). 27

**Fig. 7:**
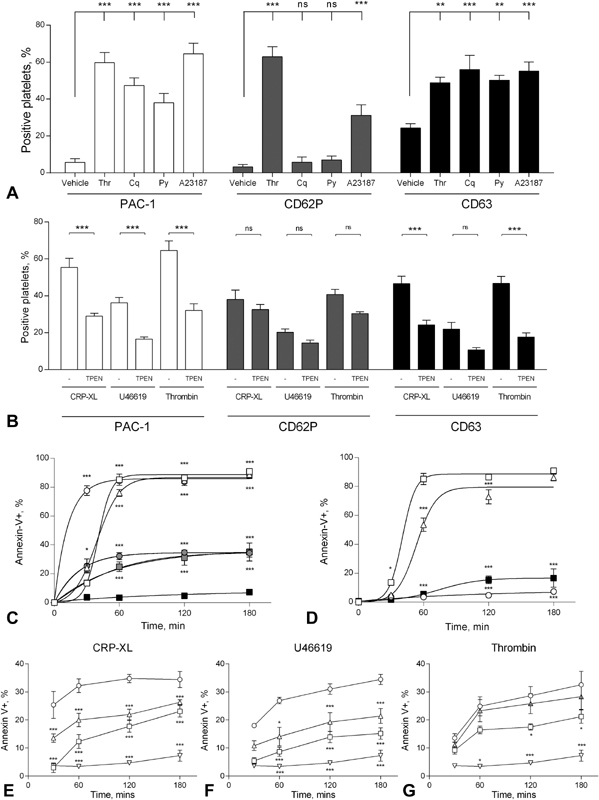
Increasing platelet [Zn ^2+^ ] _i_ using Zn ^2+^ ionophores increases platelet activation markers. ( **A** ) Washed platelet suspensions were stimulated by thrombin (Thr, 1 U/mL), clioquinol (Cq) (300 µM), pyrithione (Py) (300 µM) or A23187 (100 µM) and changes of PAC-1 (white), CD62P (grey) and CD63 (black) binding were obtained after 60 minutes. ( **B** ) Washed platelet suspensions were stimulated by CRP-XL (1 µg/mL), U46619 (10 µM) or thrombin (1 U/mL), following pre-treatment with TPEN (50 µM), and changes of PAC-1 (white), CD62P (grey) and CD63 (black) binding were obtained after 60 minutes. ( **C** ) Washed platelet suspensions were treated with Ca ^2+^ or Zn ^2+^ ionophores, or conventional platelet agonists, prior to analysis of annexin-V binding by flow cytometry. □ Clioquinol (300 µM), ▵ pyrithione (300 µM), ○ A23187 (300 µM), • CRP (1 µg/mL), ▪ thrombin, (1 U/mL), ▪ vehicle (DMSO). ( **D** ) Platelet suspensions were pre-treated with the caspase inhibitor Z-VAD (▵, 1 µM), the Zn ^2+^ chelator, TPEN (▪, 25 µm) or vehicle (□) prior to stimulation with clioquinol (300 µM). ○ Unstimulated platelets. Changes in the percentage of platelets binding to annexin-V were recorded. Washed platelets suspensions were pre-treated with Z-VAD (1 µM), or TPEN (50 µM) prior to stimulation with conventional agonists, CRP-XL (1 µg/mL, **E** ), U46619 (10 µM, **F** ) or thrombin (1 U/mL, **G** ). Changes in annexin-V binding were monitored using flow cytometry. ○ Vehicle, □ Z-VAD (1 µM), ▵ TPEN (50 µM), ▿ DMSO (no agonist). Data are means ± standard error of the mean (SEM) of at least 3 independent experiments. Significance is denoted as *** *p* < 0.001, ** *p* < 0.01 or * *p* < 0.05.

These data indicate that increases in [Zn ^2+^ ] _i_ initiate platelet activation events, such as shape change and aggregation. To better understand the extent to which changes in [Zn ^2+^ ] _i_ regulate platelet activation, the influence of Cq treatment on conventional markers of platelet activation was investigated. In a similar manner to thrombin and A23187, Cq or Py stimulation (300 µM) substantially increased platelet PAC-1 binding (59.7 ± 5.5, 64.5 ± 5.8, 47.3 ± 4.1 and 37.8 ± 5.0%, respectively, *p* < 0.05, Fig. 7A ), consistent with earlier observations correlating Cq stimulation with aggregation ( Fig. 4 ), and supportive of a role for [Zn ^2+^ ] _i_ in α _IIb_ β _3_ activation. Cq or Py increased CD63, but not CD62P externalization (55.9 ± 7.8 and 5.7 ± 2.8%, respectively, following Cq stimulation, and 50.2 ± 2.6 and 6.9 ± 2.2% following Py stimulation, Fig. 7A ) indicating that increases in [Zn ^2+^ ] _i_ initiate dense, but not α granule, secretion. This differed from both thrombin (CD62P: 62.9 ± 5.5%, CD63: 48.8 ± 3.0%) and A23187 (CD62P: 31.1 ± 5.7%, CD63: 55.1 ± 5.0%), which also regulate α and dense granule release.

Further experiments were performed to assess the influence of [Zn ^2+^ ] _i_ on agonist-evoked changes in platelet activatory markers. TPEN reduced increases of PAC-1, or CD63 binding in response to CRP-XL (1 µg/mL, from 55.4 ± 4.9 to 29.0 ± 1.5% for PAC-1 binding, and from 46.4 ± 4.0 to 24.2 ± 2.5% for CD63 binding, *p* < 0.05), U46619 (10 µM, from 36.2 ± 2.8 to 16.5 ± 1.2% for PAC-1 binding, and from 21.9 ± 3.6 to 10.7 ± 1.3% for CD63 binding, *p* < 0.05) or thrombin (1 U/mL, from 64.6 ± 5.2 to 32.1 ± 3.6% for PAC-1 binding, and from 46.8 ± 3.8 to 17.6 ± 2.3% for CD63 binding, *p* < 0.05), but had no effect on agonist-evoked CD62P increases ( Fig. 7B ). This provides further support for a role of [Zn ^2+^ ] _i_ in differentially regulating platelet granule secretion.

Extracellular Zn ^2+^ signalling and agonist-induced changes in [Zn ^2+^ ] _i_ have both been linked to apoptosis and related responses in nucleated cells. 28 29 30 31 However, the role of Zn ^2+^ in PS exposure during platelet activation has yet to be studied. To investigate the influence of [Zn ^2+^ ] _i_ on PS exposure, platelets were treated with ionophores, and annexin-V binding was quantified in real time. Increasing platelet [Zn ^2+^ ] _i_ with Cq (300 µM) resulted in a concurrent increase in annexin-V binding. PS exposure evolved more slowly with Zn ^2+^ ionophore treatment than A23817, but reached similar plateau levels (90.0 ± 0.9 and 88.6 ± 2.7% for Cq and A23187, respectively, Fig. 7C ), indicating that most platelets in the population were annexin-V positive. This differed in responses to conventional agonists, thrombin and CRP-XL, which induced PS exposure in a sub-set of platelets (35.0 ± 6.2 and 34.4 ± 6.2%, respectively). Cq-induced annexin-V binding was sensitive to TPEN (6.6 ± 6.3% positive platelets at 60 minutes) confirming a role for Zn ^2+^ . Furthermore, pre-treatment with the caspase inhibitor, Z-VAD, abrogated Cq-induced PS exposure (53.6 ± 4.7% at 60 minutes, *p* < 0.05, Fig. 7D ). The influence of Zn ^2+^ on agonist-evoked annexin-V binding was also investigated. Consistent with the findings of Cohen et al, 32 we observed a reduction in agonist-evoked PS exposure in the presence of Z-VAD (1 µM) (from 34.4 ± 2.9 to 23.1 ± 2.0% following stimulation with 1 µg/mL CRP-XL, from 24.4 ± 1.8 to 15.2 ± 2.0% following stimulation with 10 µM U46619 and from 32.5 ± 4.8 to 21.2 ± 2.4% following stimulation with 1 U/mL thrombin, Fig. 7E – G , *p* < 0.05). Similar reductions of annexin-V binding in TPEN-treated platelets were observed following stimulation by CRP-XL (26.3 ± 0.9%, *p* < 0.05), or U46619 (21.4 ± 2.7%, *p* < 0.05). However, TPEN did not affect thrombin-mediated annexin-V binding (28.3 ± 4.6%, ns). These data are consistent with a role for Zn ^2+^ in agonist-evoked PS exposure.

## Discussion

The role of Zn ^2+^ as a secondary signalling molecule has received little research interest, possibly owing to its relatively low resting cytosolic levels (pM, compared with nM concentrations of Ca ^2+^ ). Zn ^2+^ is present in granules of nucleated cells, and in platelet α granules. It also associates with thiol-containing proteins such as metallothioneins, which are also present in platelets. 33 The transition between protein- or membrane-bound Zn ^2+^ and labile Zn ^2+^ in the cell cytosol has been demonstrated in multiple cell systems, and increases in labile [Zn ^2+^ ] _i_ have been correlated with phenotypic changes. Here, we show for the first time that agonist-evoked stimulation of platelets *in vitro* results in increases of [Zn ^2+^ ] _i_ . While requiring further confirmation, such behaviour is consistent with a role of Zn ^2+^ as a secondary messenger. Zn ^2+^ fluctuations were apparent in the presence of extracellular CaCl _2_ , supporting a physiological role for this effect. We confirm the nature of the fluorescent signal using the high affinity Zn ^2+^ chelator TPEN. TPEN was also used to probe the role of Zn ^2+^ in functional responses to agonist stimulation. Owing to its affinity for Zn ^2+^ , use of TPEN here is not only likely to abrogate agonist-evoked increases in [Zn ^2+^ ] _i_ , but could also strip metalloproteins of Zn ^2+^ co-factors. 34 Thus, conclusions drawn from the use of TPEN may not only reflect abrogation of agonist-evoked [Zn ^2+^ ] _i_ increases. [Zn ^2+^ ] _i_ increases were observed in platelets following stimulation via GpVI and TP, but not via PAR, indicating that different signalling pathways link to [Zn ^2+^ ] _i_ release. Signalling via GpVI differs from that of TP or PAR G-protein-coupled receptors, in that it results in tyrosine phosphorylation of platelet proteins (such as Syk and LAT), leading to activation of PI3K and PLCγ2. Conversely, PAR and TP signal through G-protein-dependent routes to activate Rho-GEF and PLCβ. It is likely that [Zn ^2+^ ] _i_ increases are regulated by signalling proteins that are not shared by GpVI and thrombin pathways. However, the different outcomes following PAR and TP-dependent signalling are harder to reconcile, as both receptors couple to similar signalling pathways that involve Gα _12/13_ and Gα _q_ .

We show that the platelet redox state effects [Zn ^2+^ ] _i_ fluctuations in a similar manner to nucleated cells. 35 36 CRP-XL- and U46619-evoked elevations of [Zn ^2+^ ] _i_ were sensitive to antioxidants, and could be enhanced by H _2_ O _2_ . Zn ^2+^ binding to thiols (e.g. metallothioneins) is redox-sensitive and changes of redox state lead to release of Zn ^2+^ into the labile pool in nucleated cells. 37 Given that modulation of the platelet redox state led to a rapid and sustained rise of [Zn ^2+^ ] _i_ , it is possible that platelet Zn ^2+^ -binding proteins represent a store for these cations. Interestingly, Ca ^2+^ signalling was unaffected by redox changes, suggesting that these ions are differentially regulated. Indeed, the predominant Ca ^2+^ store is the dense tubular system, which performs a similar role to the endoplasmic reticulum in nucleated cells. It is therefore likely that intra-platelet Zn ^2+^ is stored by Zn ^2+^ -binding proteins and becomes liberated upon agonist stimulation. However, we did not observe increases of [Zn ^2+^ ] _i_ following thrombin stimulation, which has been shown to induce similar levels of ROS activation as collagen activation. 18 38 One possible explanation could be that the larger Ca ^2+^ signal generated by thrombin negatively regulates Zn ^2+^ release.

We examined the influence of [Zn ^2+^ ] _i_ on activatory processes using membrane permeable Zn ^2+^ -specific ionophores, Py and Cq, which have been widely used to model increases in [Zn ^2+^ ] _i_ . Stimulation with either ionophore resulted in increases in [Zn ^2+^ ] _i_ , with a greater signal obtained with Cq. Neither ionophore produced increases in Fluo-4 fluorescence, indicating a negligible affinity for [Ca ^2+^ ] _i_ . Conversely, stimulation with the Ca ^2+^ ionophore A23187 produced rapid increases in [Ca ^2+^ ] _i_ , but did not affect [Zn ^2+^ ] _i_ . Investigation of cation responses in cells depends heavily on the specificity of reagents for their cognate ions. By showing that A23187 initiates a Ca ^2+^ response which is not detected by Fz-3, we demonstrate that Fz-3 fluorescence increases are directly attributable to changes in [Zn ^2+^ ] _i_ , and are not influenced by [Ca ^2+^ ] _i_ . This is further supported by our observation that TPEN does not affect Fluo-4 fluorescence, which also provides evidence that agonist-evoked Ca ^2+^ signalling does not depend on [Zn ^2+^ ] _i_ signals. This observation raises questions about the relative roles of Ca ^2+^ and Zn ^2+^ in platelet activation, as both target similar proteins, including PKC, calmodulin and CamKII. 4 Unlike agonist stimulation, ionophore-induced [Zn ^2+^ ] _i_ increases were not sensitive to anti-oxidant treatment. Furthermore, the extent of [Zn ^2+^ ] _i_ following ionophore stimulation was greater than that observed for agonists, indicating that ionophores liberate Zn ^2+^ from stores that are not accessible to agonist-evoked signalling mechanisms. Such stores could include α granules, which are known to contain Zn ^2+^ . 20 Our use of ionophores here to model [Zn ^2+^ ] _i_ increases while providing information on Zn ^2+^ -dependent mechanisms, is therefore unlikely to fully represent the physiological situation.

Cytoskeletal re-arrangements are primary steps in platelet activation. Zn ^2+^ ionophore stimulation resulted in a demonstrable shape change, which was abrogated following Cyt-D treatment, verifying it as a biological, rather than chemical, response. Furthermore, platelet spreading on fibrinogen was abrogated following [Zn ^2+^ ] _i_ chelation. While not correlating [Zn ^2+^ ] _i_ fluctuations with shape change, these data provide support for a role of Zn ^2+^ in activation-dependent cytoskeletal re-arrangements. Zn ^2+^ is an important regulator of the cytoskeleton in nucleated cells. 39 40 Zn ^2+^ regulates tubulin polymerization leading to nuclear transport of transcription factors in neuronal cells, 41 and has been shown to regulate the actin cytoskeleton, focal adhesion dynamics and cell migration in PC-3 and HeLa cells, 35 where Zn ^2+^ chelation supresses filopodia formation and results in the loss of stress fibres. Conversely, treatment with Py increased filopodia formation, supressed stress fibres and decreased the number and size of focal adhesions. 35 Thus, Zn ^2+^ is likely to play similar important roles in platelet cytoskeletal re-arrangements. We show that raising [Zn ^2+^ ] _i_ results in increases in MLC phosphorylation. MLCK is canonically activated via Ca ^2+^ -mediated activation of calmodulin. 42 As other calmodulin-dependent kinases have been shown to be modulated by Zn ^2+^ , it is possible that Zn ^2+^ is able to substitute for Ca ^2+^ , initiating MLCK activation. 43 Absence of phosphorylation of VASP indicates that increases in [Zn ^2+^ ] _i_ do not influence the activity of cyclic nucleotide-dependent kinases such as PKG or PKA.

Ionophore-induced elevation of [Zn ^2+^ ] _i_ increased PAC-1 binding, supporting our aggregometry data ( Fig. 4 ), and supportive of role for Zn ^2+^ in regulating α _IIb_ β _3_ activity ( Fig. 6 ). Interestingly, [Zn ^2+^ ] _i_ increases resulted in the externalization of CD63, but not CD62P, supporting a role for Zn ^2+^ in regulating α, but not dense granule release. Further experiments using TPEN in conjunction with conventional platelet agonists provides support for a role for [Zn ^2+^ ] _i_ in α _IIb_ β _3_ activation and dense granule secretion, but not α granule secretion ( Fig. 7B ). Distinct signalling pathways contribute to differential release of α and dense granules, and while the exact mechanism is poorly understood, our work provides evidence for a role for Zn ^2+^ in these processes. 44 45 While these studies show that Zn ^2+^ fluctuations correlate with platelet behaviour, it should be noted that the physiological relevance of the ionophore-evoked [Zn ^2+^ ] _i_ rises are unclear and that further work will be required to establish the significance of Zn ^2+^ -dependent secondary signalling *in vivo* . Upon stimulation with conventional agonists, a sub-set of platelets adopt pro-coagulant phenotypes, elevating [Ca ^2+^ ] _i_ and externalizing PS. Extracellular Zn ^2+^ signalling, agonist-induced changes in [Zn ^2+^ ] _i_ and Zn ^2+^ ionophore treatment have all been linked to apoptosis and related responses in nucleated cells. 30 31 46 47 48 49 50 Here, we demonstrate that ionophore or agonist-evoked increases in platelet [Zn ^2+^ ] _i_ results in PS exposure, consistent with the development of a pro-coagulant phenotype. Interestingly, while CRP-XL and U46619 evoked PS exposure was sensitive to Zn ^2+^ chelation, thrombin stimulation was not. This provides further support for a role of Zn ^2+^ following GpVI and TPα signalling, but not via PARs. Unlike conventional agonists, Cq stimulation resulted in PS exposure in a majority of platelets. This may indicate that agonist-evoked Zn ^2+^ signals are stimulated in only a sub-set of platelets, which then proceed to become pro-coagulant. As previously shown ( Fig. 3 ), Cq stimulation did not induce increases in [Ca ^2+^ ] _i_ , so Cq-dependent PS exposure is independent of [Ca ^2+^ ] _i_ . Platelet PS exposure has been attributed to both caspase 3-dependent and independent mechanisms. 51 52 Cq-dependent PS exposure is partially abrogated by Z-VAD pre-treatment suggesting a partial role for caspase activity in this process.

In conclusion, this study provides the first evidence for agonist-evoked increases of [Zn ^2+^ ] _i_ in platelets. While requiring further confirmation, such behaviour is consistent with a role of Zn ^2+^ as a secondary messenger. Increases in [Zn ^2+^ ] _i_ are sensitive to the redox state, indicative of a role for redox in agonist-evoked Zn ^2+^ signalling. Modelling increases of [Zn ^2+^ ] _i_ using Zn ^2+^ -specific ionophores reveal a functional role for [Zn ^2+^ ] _i_ in platelet activatory changes. [Zn ^2+^ ] _i_ signalling contributes to key activation-related platelet responses, including shape change, α _IIb_ β _3_ activation and granule release. The mechanism by which Zn ^2+^ affects these processes is currently unknown, but could be attributable to changes in activity of Zn ^2+^ -binding enzymes. These data indicate a hitherto unknown role for labile [Zn ^2+^ ] _i_ during platelet activation, which has implications for our understanding of signalling responses in platelets. While this work does not address the physiological relevance of this process, a better understanding of Zn ^2+^ signalling may be of significance to the role of platelets in thrombotic disorders such as heart attack and stroke.

Furthermore, as they are readily available primary cells, platelets could be used as a model to better understand Zn ^2+^ signalling in other mammalian cells.

## Notes

**What is known about this topic?**

- Zinc is an intracellular secondary messenger in nucleated cells.
- Agonist-dependent fluctuations of zinc in platelets, leading to functional changes, have yet to be demonstrated.

**What does this paper add?**

- Intra-platelet zinc increases in concentration following agonist stimulation.
- Increases in zinc regulate activatory processes, including aggregation, shape change and PS exposure.
- This is the first work to demonstrate a role for zinc in agonist-dependent signal transduction in platelets.
