# Supplementary material for: Agonist-Evoked Increases in Intra-Platelet Zinc Couple to Functional Responses
Source: Thromb Haemost. 2018 Dec 31;119(1):128–39. doi: 10.1055/s-0038-1676589 (PMC6327715; doi:10.1055/s-0038-1676589)
Supplement: Supplementary file 1 — Supplementary Material [file 10-1055-s-0038-1676589-s180454.pdf]

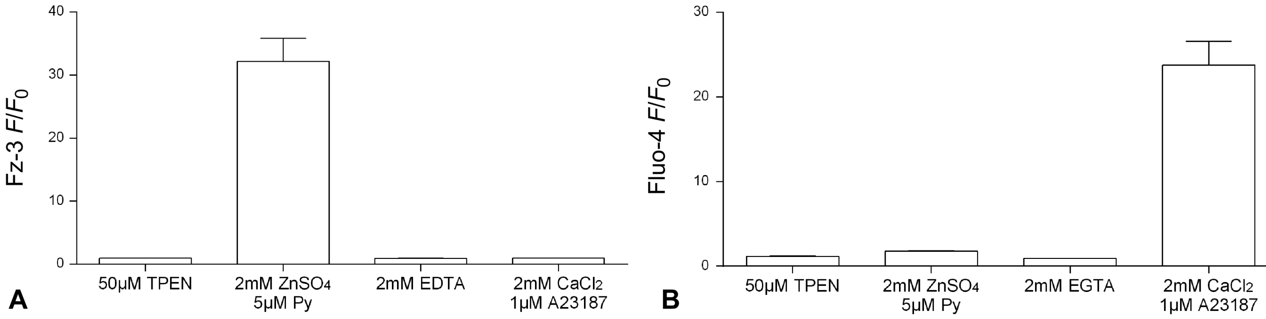

Supplementary Fig. S1 Calibration of fluorophores. Washed Fz-3 (**A**) or Fluo-4 (**B**) loaded platelet suspensions were treated for 15 minutes with Zn^{2+} or Ca^{2+} ionophores in the presence of 2 mM $CaCl_2$ or $ZnSO_4$, to determine R_{max} values. Fz-3 R_{min} was obtained from platelets pre-treated for 15 minutes with 50 μM TPEN, while R_{max} was obtained following co-incubation with 5 μM pyrithione (Py) with 2 mM $ZnSO_4$ for 15 minutes. Fluo-4 R_{min} was obtained from platelets pre-treated for 15 minutes with 2 mM EGTA in the absence of extracellular Ca^{2+}, while R_{max} was obtained following pre-incubation with 1 μM A23187 in the presence of 2 mM $CaCl_2$.

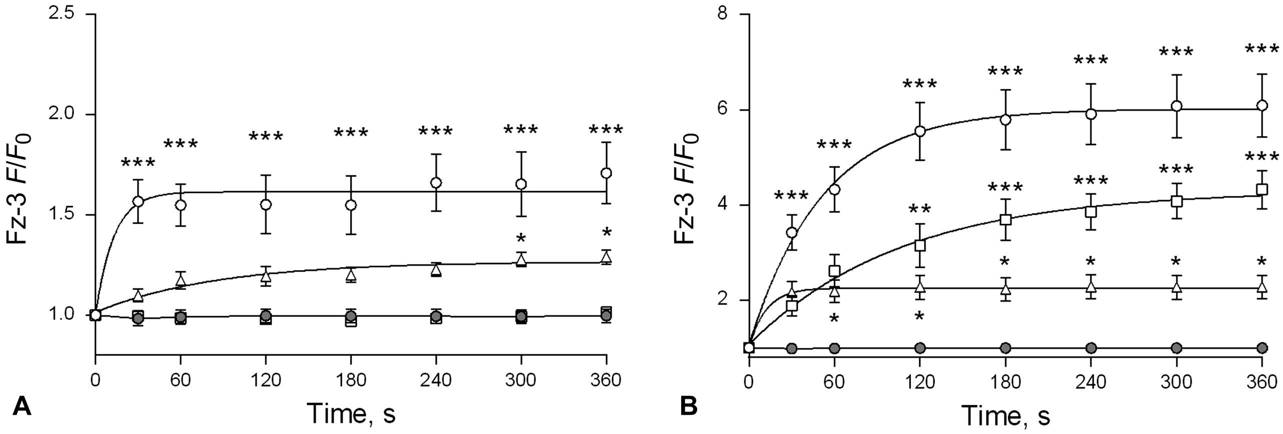

Supplementary Fig. S2. Extracellular calcium does not significantly affect agonist- or ionophore-evoked $[Zn^{2+}]_i$ fluctuations. Washed, Fz-3 loaded platelets suspended in Tyrode's buffer supplemented with 2 mM $CaCl_2$ were stimulated with platelet agonists (**A**); CRP-XL (1 μg/mL, ○), U46619 (10 μM, ○), thrombin (1 U/mL) or ionophores (**B**); clioquinol (□, 300 μM), pyrithione, (△, 300 μM), A23187 (○, 300 μM), during which changes in fluorescence were monitored. ● vehicle control (DMSO). Data are mean ± standard error of the mean (SEM) from at least 4 independent experiments. Significance is denoted as $^{***}p < 0.001$, $^{**}p < 0.01$ or $^{*}p < 0.05$.
